# Acute Oral Toxicity and Genotoxicity of Polysaccharide Fraction from Young Barley Leaves (*Hordeum vulgare* L.)

**DOI:** 10.3390/foods9060809

**Published:** 2020-06-19

**Authors:** Chang-Won Cho, Young-Ran Song, Won-Chul Lim, Youn-Hwan Hwang, Young Kyoung Rhee, Jae Woong Choi, Kyung-Tae Lee, Hee-Do Hong

**Affiliations:** 1 Research Group of Traditional Food, Korea Food Research Institute, Wanju-gun, Jeollabuk-do 55365, Korea; cwcho@kfri.re.kr (C.-W.C.); songyr28@efact.or.kr (Y.-R.S.); 07934@kfri.re.kr (W.-C.L.); ykrhee@kfri.re.kr (Y.K.R.); choijw@kfri.re.kr (J.W.C.); 2 Herbal Medicine Research Division, Korea Institute of Oriental Medicine, Daejeon 34054, Korea; hyhhwang@kiom.re.kr; 3 Korean Convergence Medicine Major KIOM, University of Science & Technology (UST), Daejeon 34054, Korea; 4 Department of Pharmaceutical Biochemistry, College of Pharmacy, Kyung Hee University, Seoul 02447, Korea; ktlee@khu.ac.kr

**Keywords:** *Hordeum vulgare* L., polysaccharides, toxicity

## Abstract

Polysaccharides isolated from various plants are considered precious bioactive materials owing to their potent biological activities. Previously, we prepared a polysaccharide fraction (BLE0) isolated from young barley leaves (*Hordeum vulgare* L.), demonstrating its anti-osteoporotic and immunostimulatory activities. However, data regarding BLE0 toxicity is lacking. To establish its safety, in vitro genotoxicity (chromosomal aberration and bacterial reverse mutation assays) and acute oral toxicity assays were conducted. In the in vitro genotoxicity assays, bacterial reverse mutation and chromosomal aberration assays showed that BLE0 possessed no mutagenicity or clastogenicity. Furthermore, the median lethal dose (LD_50_) of BLE0 was higher than 5000 mg/kg in female and male Sprague-Dawley (SD) rats and no adverse effects were observed in terms of mortality and abnormal changes in clinical signs (body weight and necropsy). Based on these results, BLE0 was found to be safe with regards to genotoxicity under our test conditions, demonstrating no acute oral toxicity up to 5000 mg/kg in SD rats.

## 1. Introduction

Barley (*Hordeum vulgare* L.) is an edible crop belonging to the family *Poaceae*, and is mainly used as a food source in East Asia, including Korea [1]. Barley is known to contain various functional ingredients such as lutonarin, saponarin, β-glucan and various policosanols [2,3]. Particularly, it has been reported that after seeding, young barley leaves (15–20 cm in size) contain a large amount of superoxide dismutase and vitamin C, which decompose free radicals [4]. Additionally, young barley leaves contain a large amount of saponarin, which demonstrates improved liver function as well as inhibits α-glucosidase, a diabetes-related enzyme, among polyphenol compounds [5,6]. Studies evaluating the various biological activities of the young barley leaf, such as antioxidant, antidiabetic, antidepressant and hypolipidemic effects, have been reported [1,2,7,8,9].

To date, most physiological studies using the young barley leaf have been conducted with solvent extracts containing small molecules, such as saponarin, as the main active ingredient. In addition to plant-derived bioactive small molecules, various biological properties (immune-enhancing, anti-inflammatory, antiviral, and anticancer activities) of plant-derived polysaccharides, major high-molecular-weight plant substances, have been recently reported [10,11]. According to recent research trends, we purified the pectinase-treated polysaccharide fraction (BLE0) from young barley leaves. Furthermore, we reported immune-enhancing and antiosteoporotic activities of BLE0, revealing the detailed structure and molecular mechanisms underlying its immunostimulatory activities [12,13,14,15,16].

The development of medicines and health-functional foods using natural products is actively advancing and, accordingly, the scientific basis for the safety of each natural product is considered important [17]. Although most extracts derived from natural products are safe, some have known toxicities [18,19,20]. Thus, when using natural products as medicines, or as ingredients in functional foods, safety evaluation is essential.

Therefore, in this study, the in vivo acute toxicity and in vitro genotoxicity of BLE0 were evaluated to establish preliminary safety data to enable the development of health-functional foods derived from BLE0.

## 2. Materials and Methods

### 2.1. Preparation of BLE0

Pilot-scale manufactured BLE0 was obtained from young barley leaves according to a previously reported method with minor modifications [16]. Samples of young barley leaves, cultivated in Naju (Jeollanam-do, Korea) in 2017, were harvested 2 weeks after seeding. The polysaccharides (BLE0) from barley leaves were prepared by enzyme-assisted extraction using commercial pectinase Plantase MAX^®^ from *Aspergillus niger* (Vision, Seongnam, Korea). The dried barley leaves (10 kg) were minced and suspended in 300 L of distilled water (solid/liquid ratio, 1:30 *w*/*v*). The pH of the suspension was adjusted to an optimal pH range of 4.5–5.0. Enzyme digestion and extraction were conducted at 50 °C for 3 days in a circulation extraction tank in which 1.5% of pectinase (*v*/*w*) was added twice. After the enzymatic hydrolysis process, the extract from the enzyme hydrolyzate was filtered through a 0.5 μm depth cartridge filter (Pall Corporation, Port Washington, NY, USA), and concentrated to the final soluble solids content of 5.0°Brix at reduced pressure. After the gradual addition of 2.5 times 95% (*v/v*) cold ethanol to the aqueous extract, the extract was stored overnight at −20 °C to precipitate crude polysaccharides. The precipitate was collected by centrifugation, dissolved in water (solid/liquid ratio, 1:7 *w*/*v*) at 50 °C and then dried using a pilot-scale spray dryer (SD Pilot 2010; Ein system, Seoul, Korea) to yield BLE0. Spray drying was performed with an inlet air temperature of 190 °C and an outlet air temperature of 90 °C while maintaining the temperature of the sample sludge at 50 °C. The spray dryer sample feed velocity was 10 L/h. No additives were used. To prevent cross-contamination between samples, the drying chamber was washed with detergent and hot water and a run without sample was performed between samples.

### 2.2. Chemical Composition of BLE0

The yield (%) was determined as dry weight of the obtained polysaccharide sample relative to the weight of young barley leaves. Total sugar content was determined using the phenol-sulfuric acid method (standard: glucose) [21] and acidic sugar content was determined by the carbazole sulfuric acid method (standard: _D_-galacturonic acid) [22]. Protein content was determined by the Bradford method (standard: bovine serum albumin) [23] and 2-Keto-3-Deoxy-D-mannooctanoic acid (KDO)-like materials were measured by a modified thiobarbituric acid-positive method (standard: KDO) [24]. The monosaccharide composition of BLE0 was analyzed using high-performance anion-exchange chromatography with pulsed amperometric detection (HPAEC-PAD) in an ICS-5000 chromatography system (Dionex, Sunnyvale, CA, USA) after polysaccharides were degraded into monosaccharides by acid hydrolysis with 2 M trifluoroacetic acid in a boiling water bath for 4 h. The hydrolysate was adjusted to pH 6.0, diluted with ultrapure water and then filtrated through 0.22 μm hydrophilic membranes. Table 1 shows the experimental conditions for HPAEC-PAD analysis. Neutral sugars were eluted with 18 mM NaOH, and uronic acids were eluted with 100 mM NaOAc in 100 mM NaOH (isocratic elution).

### 2.3. Bacterial Reverse Mutation Test

To confirm the mutagenicity of BLE0, a bacterial reverse mutation assay was performed based on the Organization for Economic Cooperation and Development (OECD) Test Guideline 471 (TG471) [25] and the guidelines of the National Institute of Food and Drug Safety Evaluation (NIFDS) of Korea (No. 2014-136) [26]. Histidine-requiring strains (*Salmonella typhimurium* TA98, TA100, TA1535, and TA1537) and a tryptophan-requiring strain (*Escherichia coli* WP2*uvrA*) were used. As a metabolic activation system, S9 mix (Moltox., Boone, NC, USA: 50 μL of rat liver S9 fraction in 1 mL of S9 mix, 5 µM glucose-6-phosphate, 8 µM MgCl_2_·6H_2_O, 33 µM KCl, 4 µM NADPH, 4 µM NADH, 100 µM sodium phosphate buffer, pH 7.4) was used. For all strains, colony counts were measured with and without the metabolic activation system. Sodium azide (SA), 2-Nitrofluorene (2-NF), acridine mutagen ICR 191 (ICR-191), 4-Nitroquinoline-1-oxide (4NQO), 2-Aminoanthracene (2-AA), and benzo[a]pyrene (B[a]P) were used as positive controls. All positive controls were obtained from Sigma-Aldrich (St. Louis, MO, USA).

### 2.4. In Vitro Mammalian Chromosomal Aberration Assay

A BLE0-induced chromosomal aberration test was performed based on OECD guideline TG473 [27] and guidelines of NIFDS of Korea (No. 2014-136) [26] using Chinese hamster lung fibroblast (CHL/IU) cells. CHL/IU cells (CRL-1935) were procured from the American Type Culture Collection (Manassas, VA, USA). Cells were maintained in minimum essential medium (Gibco BRL, Grand Island, NY, USA) containing 10% fetal bovine serum. Cells were cultured by adding test or control substances in the absence and presence of an S9 metabolic activation system (S9 mix: 200 μL of rat liver S9 fraction in 1 mL of S9 mix, 5 µM glucose-6-phosphate, 8 µM MgCl_2_·6H_2_O, 33 µM KCl, 5 µM NADP). A solution of colchicine (final concentration 1 μM) was added approximately 2 h before the end of the culture. After incubation, the culture medium containing metaphase cells was centrifuged at 200 g for 5 min followed by the addition of a fixing solution (glacial acetic acid: methanol = 1:3 *v/v*). The cell suspension was placed on a glass slide to prepare a slide sample. After drying, the samples were stained for 20 min with a Giemsa staining solution (5%) and analyzed for chromosomal abnormalities under a 1000-fold microscope.

### 2.5. Acute Oral Toxicity Test

For acute oral toxicity, 6-week-old specific-pathogen-free female and male Sprague-Dawley (SD) rats were procured from Daehan Biolink (Eumseong, Korea). An irradiation-sterilized pellet diet for lab animals (LabDiet 5L79; Charles River, Wilmington, MA, USA) and groundwater (UV sterilized and filtered) were provided ad libitum. All animal experiments were approved by the Institutional Animal Care and Use Committee of Croen Inc. (19R073; Suwon, Korea).

A single-dose acute oral toxicity assay was performed based on the guidelines (No. 2017-71) of the NIFDS of Korea [28]. SD rats were selected as they are extensively used in general toxicity studies, presenting abundant comparable evidence [29]. The experimental animals included 22 males and 22 females, weighing 149.4–171.0 g and 137.0–153.3 g, respectively. After animal acquisition, a 7-day acclimatization period was allowed. Body weight was measured from the day before the start of BLE0 administration, recorded as 233.3–263.4 g in males and 168.6–195.7 g in females (supplementary material Table S1). The rats were randomly divided to a control group and three test groups (1250, 2500, and 5000 mg/kg). A total of 40 animals were used (5/sex/group). In the dose-range finding study (*n* = 4), no dead animals were observed at 5000 mg/kg. Hence, 5000 mg/kg was set as the high-dose group, 2500 mg/kg as the medium-dose group and 1250 mg/kg as the low-dose group. The negative control group was administered sterile distilled water (Lot No.: V3T7B21: Daihan Pharmaceutical, Seoul, Korea) as a vehicle control. After an overnight fast, BLE0 was administered as a single oral dose using a feeding needle (20 mL/kg), and food was provided 3 h after administration. All experimental animals were observed for general clinical signs as well as weight measurements. On the day of administration, clinical signs were observed every hour until 4 h after administration, and once every 14 days thereafter. Additionally, body weights were measured 1, 3, 7, and 14 days after administration. On the 14th day after administration, all organs were visually examined following animal autopsies after CO_2_ euthanasia.

### 2.6. Statistical Analysis

Experimental data are expressed as mean ± standard deviation. For animals, body weight data were statistically analyzed by one-way analysis of variance. For the in vitro chromosomal aberration test, Fisher’s exact test was used to compare data between the negative control group and the treatment groups or the positive control group. The significance level was set at *p* < 0.05. All statistical analyses were conducted using SPSS version 24 (SPSS Inc., Chicago, IL, USA).

## 3. Results

### 3.1. Chemical Composition of BLE0

Previously, we obtained a polysaccharide fraction (BLE0) from pectinase hydrolysate of young barley leaves with immunostimulatory and antiosteoporotic effects [12,13,14,15,16]. It was demonstrated that pectinase application was a useful method for obtaining polysaccharides with enhanced immune activity from barley leaves [12]. In this study, BLE0 was obtained from young barley leaves by utilizing pectinase-assisted extraction at a pilot-scale for commercial production. Extraction yield of BLE0 was 4.1%. Neutral sugars (79.2%) and uronic acid (18.0%) comprised the main parts of BLE0, demonstrating a high carbohydrate content (>97%) summed by neutral and acidic sugars (Table 2). Additionally, BLE0 contained protein and KDO-like materials. The sugar composition of BLE0 was further analyzed by HPAEC-PAD revealing that BLE0 was mainly composed of xylose, galactose, arabinose, glucose and galacturonic acid, in addition to fucose, rhamnose and glucuronic acid (Supplementary Material Figure S1). In terms of peak area, the high levels of arabinose, xylose, galactose and galacturonic acid indicated that BLE0 possessed arabinoxylan and rhamnogalacturonan I-rich structures, which is in accordance with previous results [14]. Additionally, the chemical and monosaccharide compositions of BLE0 were similar to those in previous results of BLE0 prepared by a laboratory-scale experiment [16], indicating stable BLE0 production from the pilot-scale process.

### 3.2. Bacterial Reverse Mutation Test

**Figure 1:**
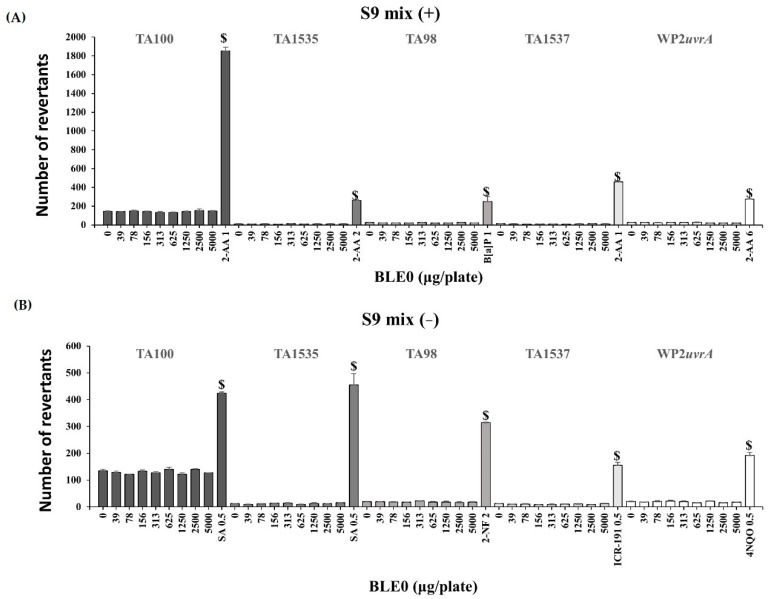
Effect of BLE0 on bacterial reverse mutation (**A**) with (+S9 mix) or (**B**) without (-S9 mix) the metabolic activation of *Salmonella typhimurium* (TA100, TA1535, TA98, and TA1537) and *Escherichia coli* (WP2*uvrA*). $, positive bacterial reverse mutation induced by positive control; SA, sodium azide; 2-AA, 2-Aminoanthracene; 2-NF, 2-nitrofluorene; ICR-191, acridine mutagen ICR 191; 4NQO, 4-Nitroquinoline-1-oxide; B[a]P, benzo[a]pyrene.

The bacterial reverse mutation assay was conducted using histidine demand test strains (*S. typhimurium*) [30] and a tryptophan demand test strain (*E. coli*) [31]. In all tested strains, no cytotoxicity was detected at BLE0 concentrations up to 5000 μg/plate. As shown in Figure 1, the mean number of colonies in the BLE0-treated groups did not show any increases, regardless of treatment with the S9 metabolic activation system in all tested strains. On the other hand, the mean revertant of the positive control for each test strain exhibited a clear increase. These results suggested that the experimental system worked well. Therefore, it was confirmed that BLE0 does not induce a reverse mutation in the evaluated bacterial strains used as a standard test method.

### 3.3. In Vitro Mammalian Chromosomal Aberration Assay

BLE0-induced chromosomal abnormalities were investigated using CHL/IU cells. BLE0 treatment was performed for 6 or 24 h and structural and numerical aberrations were observed after Giemsa staining. Structural aberrations analyzed the gap, break, exchange and the fragment of chromosome and chromatid; numerical aberrations evaluated the frequency of endoreduplication (ER) and polyploidy (PP). The frequency of structural abnormalities in the BLE0-treated and negative control cells was found to be 0%, indicating that the frequency of structural abnormalities was not increased by BLE0 administration. In contrast, in the positive control groups, structural abnormality of chromosomes increased significantly. Additionally, the frequency of numerical abnormalities induced by BLE0 did not demonstrate a significant difference from the negative control group. These results suggested that BLE0 does not cause chromosomal abnormalities (Table 3).

(1) $$\mathrm{RICC}\left(\%\right)=\frac{\mathrm{Cell}\;\mathrm{number}\;\mathrm{increase}\;\mathrm{in}\;\mathrm{treated}\;\mathrm{cultures}\;\left(\mathrm{final}-\mathrm{initial}\right)}{\;\mathrm{Cell}\;\mathrm{number}\;\mathrm{increase}\;\mathrm{in}\;\mathrm{negative}\;\mathrm{control}\;\mathrm{cultures}\;\left(\mathrm{final}-\mathrm{initial}\right)}\times 100.$$

### 3.4. Acute Oral Toxicity Test

**Figure 2:**
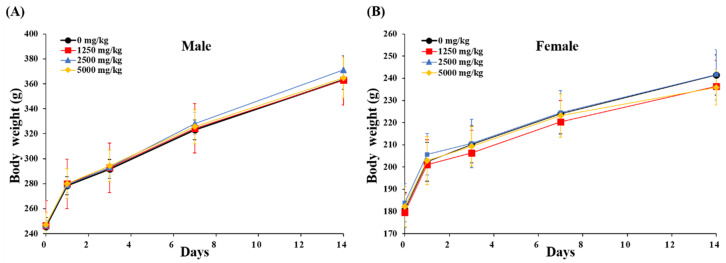
Body weight change of male (**A**) and female (**B**) rats in the single-dose oral toxicity test of BLE0. The day of administration was designated as day 0. Male and female rats were randomly divided into four groups (*n* = 5/sex/group). Data are presented as mean ± standard deviation.

To determine single-dose toxicity and the approximate lethal dose of BLE0, the test substance was orally administered once to male and female SD rats and general symptoms, weight change and deaths were observed for 14 days after administration. No animals died during the experimental period and no experimental animal demonstrated unusual general clinical features. Body weight was measured before and 1, 4, 7 and 14 days after BLE0 administration. Only normal weight gain was observed with no significant changes measured between the BLE0 treated groups and the control group (Figure 2 and Supplementary Material Table S1). On the 14th experimental day, autopsies were performed on the organs of all surviving animals with no specific findings observed in all experimental animals (data not shown). The median lethal dose (LD_50_) of BLE0 was >5000 mg/kg.

## 4. Discussion

In 2017, the market for food and medicine derived from natural products was approximately 1 trillion dollars worldwide, growing by 8–10% every year [32]. Notably, natural health-functional foods and medicines have demonstrated relatively low toxicity, with shorter development times when compared to synthetic substances [33]. Additionally, unlike synthetic materials, diverse active ingredients were found to be present in one natural product, thereby enhancing clinical effects during disease conditions [34]. Hence, research on natural products is being actively conducted in several countries. However, systematic safety evaluation of natural products used as raw materials for pharmaceuticals and functional foods is still insufficient when compared to studies evaluating efficacy. In Korea, a list of approved compounds/foods is presented under relevant regulations. However, safety evaluations are essential when new compounds/foods are introduced into the market [26]. In our preliminary studies, we elucidated the major components and structural characteristics of BLE0 [12,13]. Moreover, our previous studies showed that BLE0 possesses immune-enhancing and anti-osteoporitic activities [14,15,16]. However, safety assessment data are crucial to exclude the potential toxicities of BLE0.

Therefore, in this study, we aimed to establish preliminary safety data that would permit the development of BLE0 as a health-functional food. Accordingly, in vivo acute oral toxicity and in vitro genotoxicity tests were performed to assess the safety of BLE0.

The bacterial reverse mutation assay is a widely used method for assessing whether a test substance can induce mutations in the DNA of model microorganisms [30]. The bacterial reverse mutation test was performed to determine whether BLE0 could induce a return mutation in the *S. typhimurium* histidine-requiring strains (TA98, TA100, TA1535, TA1537) and the *E. coli* tryptophan-requiring strain (WP2*uvrA*), in the absence and presence of a metabolic activation system. As a metabolic activation system, a cofactor was added to the liver homogenate of rats induced with Aroclor-1254. Regardless of the absence or presence of the metabolic activation system, the average number of colonies (*S. typhimurium* TA98, TA100, TA1535, TA1537, and *E. coli* WP2*uvrA*) in the BLE0 treatment groups did not increase. Conversely, a positive increase in the colony count was observed in all positive control groups, suggesting that BLE0 does not cause a return mutation in the test strains used under these test conditions.

Studies regarding chromosomal DNA damage induced by test substances are essential for genotoxicity screening [35]. In this study, a chromosomal aberration test was performed to examine the clastogenicity of BLE0. Chromosomal abnormalities were identified and numbered based on the atlas of chromosome aberrations by chemicals [36]. In the BLE0 treatment groups, the frequency of abnormal metaphase expression was confirmed as 0%, with or without the metabolic activation system, and the absence of chromosomal abnormalities was verified. In contrast, in all positive controls, the frequency of abnormal mid-phase expression was significantly increased. Based on the above results it was concluded that BLE0 did not cause chromosomal abnormalities in the CHL/IU cells under the current experimental conditions. Taken together, the present genotoxicity data for BLE0 were consistent with previous reports that demonstrated no mutagenicity of the polysaccharide fraction from various plant extracts [37,38,39].

A single-dose oral administration of BLE0 did not result in abnormal changes in body weight, clinical signs, mortality or autopsy findings in SD rats. Therefore, under this acute oral toxicity experiment condition, the median lethal dose (LD_50_) of BLE0 was determined to be higher than 5000 mg/kg. Based on OECD test guidelines, BLE0 was considered nontoxic owing to the absence of deaths at a dose of 2000 mg/kg [29], thus suggesting the non-toxicity of BLE0. Previously, the polysaccharide fraction of plant extracts demonstrated no toxicity after a single-dose oral administration of 5000 mg/kg in SD rats [37,40]. The present results are in accordance with previous results.

In this study, under the given experimental conditions, BLE0 did not demonstrate in vitro genotoxicity or in vivo acute oral toxicity. However, in addition to the preliminary safety data obtained in this study, in vivo micronucleus and subchronic (28 days) oral toxicity experiments should be conducted to satisfy the overall safety requirements for the development of health-functional foods.

## 5. Conclusions

In the present study, an in vitro genetic toxicity test and a single-dose acute oral toxicity test were performed to evaluate the feasibility of using BLE0 (a bioactive polysaccharide fraction isolated from young barley leaves) as a functional food material. For genotoxicity testing, bacterial reverse mutation and in vitro chromosome aberration tests were used. In the bacterial reverse mutation assay, no significant increase in colony number was observed after BLE0 treatment in the absence or presence of an S9 metabolic activation system. Additionally, in the in vitro chromosome aberration test, no chromosomal abnormalities were induced in the BLE0 treated groups. In a single-dose acute oral toxicity test, oral administration of BLE0 (0, 1250, 2500, and 5000 mg/kg) presented no abnormal symptoms, including death or serious weight changes, in SD rats. Therefore, it was concluded that the LD_50_ of BLE0 was greater than 5000 mg/kg. Collectively, these results suggest that BLE0 does not cause genetic toxicity by inducing bacterial reverse mutations or chromosomal abnormalities and presents no acute oral toxicity in SD rats.

## Figures and Tables

**Table 1:** High-performance anion-exchange chromatography with pulsed amperometric detection (HPAEC-PAD) operating condition for sugar composition analysis.

Column: CarboPac PA1 analytical column (4 × 250 mm, Dionex) Column temperature: 30 °C Injection Volume: 25 µL Flow rate: 1.0 mL/min

**Table 2:** Chemical properties of polysaccharides manufactured from pectinase-treated *H. vulgare* leaves (BLE0).

	BLE0 ^1^
**Yield (%)**	4.1 ± 0.3 ^2^
Chemical composition (%) ^3^	
Neutral sugar	79.2 ± 0.1
Uronic acid	18.0 ± 1.4
2-Keto-3-Deoxy-Mannoctanoic acid (KDO)-like materials	2.3 ± 1.2
Protein	0.4 ± 0.0
Composition of sugar (mol%) ^4^	
Arabinose	16.9 ± 0.0
Fucose	0.8 ± 0.0
Galactose	18.5 ± 0.1
Glucose	14.8 ± 0.1
Mannose	nd ^5^
Rhamnose	5.5 ± 0.1
Xylose	29.4 ± 0.2
Galacturonic acid	11.5 ± 0.2
Glucuronic acid	2.6 ± 0.0

^1^ BLE0 was manufactured on a pilot-scale for industrial applications; ^2^ values represent the means and standard deviations on BLE0 prepared by three independent experiments; ^3^ percentage (%) of each dry material; ^4^ Mol% was calculated from detected total sugar; ^5^ nd, not detected.

**Table 3:** In vitro chromosomal aberration assay in Chinese hamster lung cells treated with BLE0.

Trt-Rec Time ^1^ (h)	S9 mix ^2^	Dose (μg/mL)	No. Aberrant Metaphase ^3^ (%)	PP+ER ^3, 4^ (%)	RICC ^5^ (%)
6–18	+	0	0(0.00)	0(0.00)	100
		31.25	0(0.00)	0(0.00)	96
		62.5	0(0.00)	0(0.00)	98
		125	0(0.00)	0(0.00)	89
		250	0(0.00)	0(0.00)	89
		500	0(0.00)	0(0.00)	93
		1000	0(0.00)	0(0.00)	83
		B[a]P 20	51(34.00) **	0(0.00)	52
6–18	−	0	0(0.00)	0(0.00)	100
		31.25	0(0.00)	0(0.00)	88
		62.5	0(0.00)	0(0.00)	87
		125	0(0.00)	0(0.00)	91
		250	0(0.00)	0(0.00)	90
		500	0(0.00)	0(0.00)	89
		1000	0(0.00)	0(0.00)	77
		4NQO 0.4	18(12.00) **	0(0.00)	78
24–0	−	0	0(0.00)	0(0.00)	100
		31.25	0(0.00)	0(0.00)	102
		62.5	0(0.00)	0(0.00)	98
		125	0(0.00)	0(0.00)	99
		250	0(0.00)	0(0.00)	92
		500	0(0.00)	0(0.00)	74
		1000	0(0.00)	0(0.00)	68
		4NQO 0.4	20(13.33) **	0(0.00)	87

^1^ Trt-Rec Time, treatment time, recovery time; ^2^ the absence (−) or presence (+) of S9 mix; ^3^ number of cells in metaphase with chromosomal aberrations; after gaps were excluded, 150 metaphase cells/culture were examined; ^4^ PP, polyploidy; ER, endoreduplication; ^5^ RICC, relative increase in cell count (Equation (1)); ** *p* < 0.01 compared to the negative control (Dose = 0 μg/mL; Fisher’s exact test).

## Supplementary Materials

The following are available online at https://www.mdpi.com/2304-8158/9/6/809/s1, Figure S1: HPAEC-PAD chromatograms showing the monosaccharide compositions of BLE0, Table S1: Body weight.
