# Supplementary material for: Acute Oral Toxicity and Genotoxicity of Polysaccharide Fraction from Young Barley Leaves (Hordeum vulgare L.)
Source: Foods. 2020 Jun 19;9(6):809. doi: 10.3390/foods9060809 (PMC7353472; doi:10.3390/foods9060809)
Supplement: Supplementary file 1 [file foods-09-00809-s001.pdf]

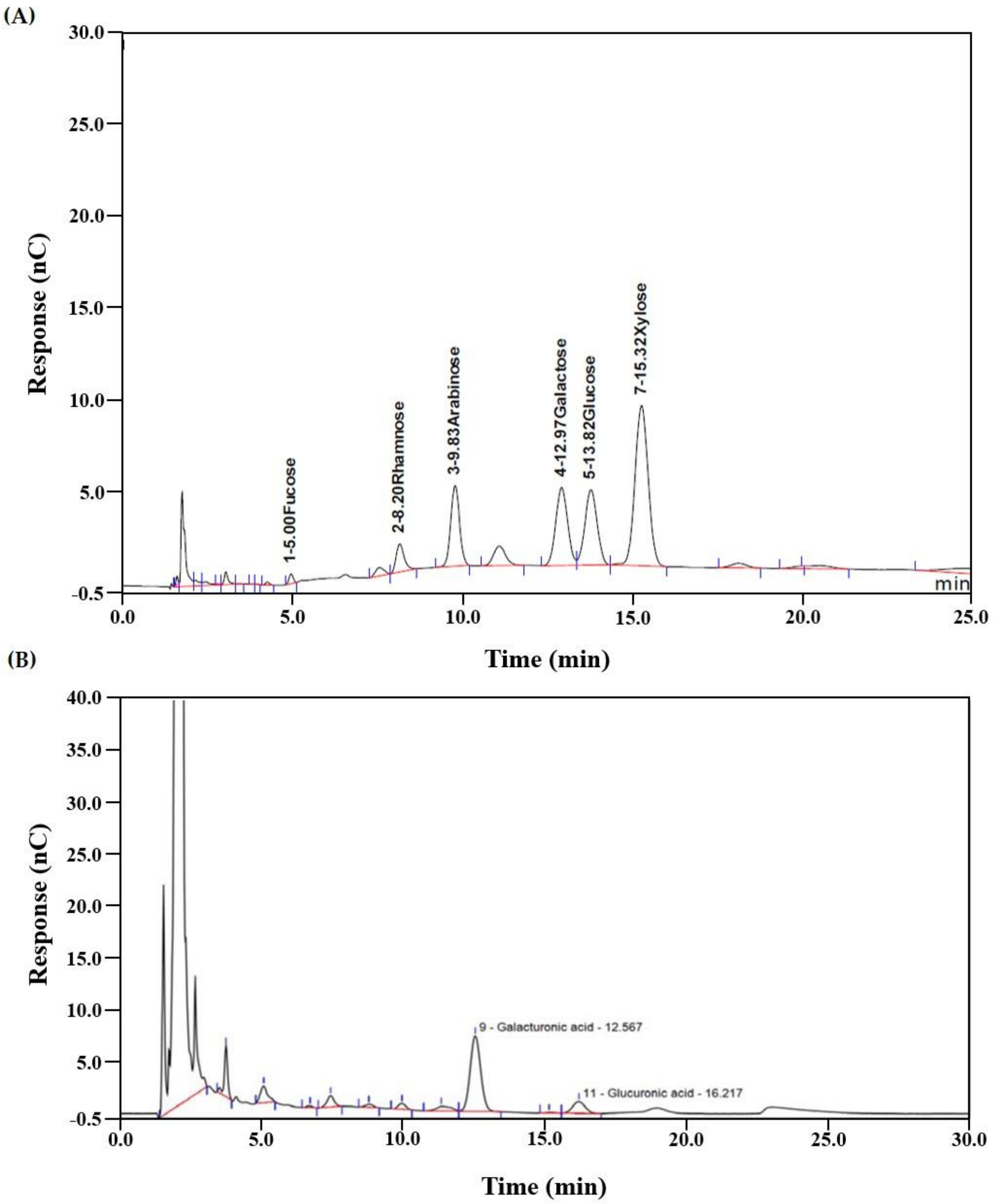

Figure S1. HPAEC-PAD chromatograms showing the monosaccharide compositions of BLE0. (**A**) Fucose (RT; 5.00 min), rhamnose (8.20 min), arabinose (9.83 min), galactose (12.97 min), glucose (13.82 min), and xylose (15.32 min) are identified as neutral sugars. (**B**) Galacturonic acid (12.567 min) and glucuronic acid (16.217 min) are identified as acidic sugars.

Table S1. Body weight (g, *n* = 5).

Group / BLE0 Dose (mg/kg)	Animal ID	Day 0	1	3	7	14	0–14 (Gain)
G1 0	1101	233.31	266.44	279.67	310.50	354.40	121.09
	1102	245.96	279.65	291.06	324.74	361.60	115.64
	1103	248.45	278.84	293.46	325.26	371.34	122.89
	1104	250.38	285.74	295.06	323.14	358.29	107.91
	1105	250.26	281.43	299.77	332.05	370.48	120.22
	Mean	245.67	278.42	291.80	323.14	363.22	117.55
	S.D.	7.14	7.21	7.49	7.85	7.47	6.02
G2 1250	1201	240.40	275.03	287.61	321.76	365.52	125.12
	1202	246.25	276.04	287.91	317.80	351.43	105.18
	1203	247.26	284.33	295.69	325.76	365.36	118.10
	1204	250.99	282.79	298.94	332.57	373.58	122.59
	1205	248.06	281.51	293.82	323.77	358.31	110.25
	Mean	246.59	279.94	292.79	324.33	362.84	116.25
	S.D.	3.89	4.16	4.95	5.47	8.36	8.38
G3 2500	1301	240.73	268.46	285.09	319.06	357.47	116.74
	1302	244.92	278.99	289.89	321.25	363.85	118.93
	1303	247.81	281.51	291.40	328.39	370.94	123.13
	1304	247.97	281.20	295.54	330.81	381.08	133.11
	1305	251.97	285.37	303.12	341.45	383.28	131.31
	Mean	246.68	279.11	293.01	328.19	371.32	124.64
	S.D.	4.17	6.38	6.78	8.86	11.02	7.31
G4 5000	1401	240.99	270.05	283.67	308.10	342.13	101.14
	1402	239.64	271.09	285.75	325.02	366.32	126.68
	1403	244.43	278.82	295.78	324.53	361.53	117.10
	1404	247.17	280.40	291.13	324.09	366.06	118.89
	1405	263.41	299.65	315.03	346.28	388.01	124.60
	Mean	247.13	280.00	294.27	325.60	364.81	117.68
	S.D.	9.57	11.90	12.53	13.58	16.33	10.05

Sex: Male.

Table S1. *Cont*

Group / BLE0 Dose (mg/kg)	Animal ID	Day 0	1	3	7	14	0–14 (Gain)
G1 0	2101	168.69	187.72	197.14	211.88	230.01	61.32
	2102	180.07	206.35	211.86	222.57	241.32	61.25
	2103	179.21	201.59	207.73	220.82	235.37	56.16
	2104	187.33	205.49	216.49	235.08	252.38	65.05
	2105	188.27	210.44	217.79	229.56	248.19	59.92
	Mean	180.71	202.32	210.20	223.98	241.45	60.74
	S.D.	7.88	8.75	8.32	8.84	9.12	3.19
G2 1250	2201	168.58	186.93	192.34	208.74	229.53	60.95
	2202	174.90	193.81	204.61	215.34	223.61	48.71
	2203	180.34	201.26	205.19	221.40	230.92	50.58
	2204	185.45	208.16	209.56	221.48	247.79	62.34
	2205	188.90	215.18	220.32	234.79	250.01	61.11
	Mean	179.39	201.07	206.40	220.35	236.37	56.74
	S.D.	8.14	11.20	10.08	9.63	11.79	6.53
G3 2500	2301	173.39	194.55	195.09	210.74	235.32	61.93
	2302	178.55	199.65	206.59	218.35	227.68	49.13
	2303	184.40	204.37	211.09	226.18	242.79	58.39
	2304	187.59	212.56	216.21	232.91	258.02	70.43
	2305	195.74	217.47	224.13	234.24	244.10	48.36
	Mean	183.93	205.72	210.62	224.48	241.58	57.65
	S.D.	8.56	9.33	10.86	9.94	11.30	9.23
G4 5000	2401	173.46	199.20	200.90	214.31	237.47	64.01
	2402	173.59	192.94	202.51	216.56	224.76	51.17
	2403	185.54	202.04	211.57	223.43	233.98	48.44
	2404	182.82	199.02	208.07	222.22	236.46	53.64
	2405	195.48	221.25	223.33	239.13	246.92	51.44
	Mean	182.18	202.89	209.28	223.13	235.92	53.74
	S.D.	9.20	10.79	8.95	9.72	7.94	6.03

Sex: Female.
